# Measurement invariance across age, gender, ethnicity, and psychopathology of the Psychotic‐Like Experiences Questionnaire for Children in a community sample

**DOI:** 10.1002/mpr.1962

**Published:** 2023-03-02

**Authors:** Lauren M. Piltz, Emma J. Carpendale, Kristin R. Laurens

**Affiliations:** ^1^ School of Psychology and Counselling Queensland University of Technology (QUT) Kelvin Grove Queensland Australia; ^2^ Department of Psychosis Studies Institute of Psychiatry, Psychology, and Neuroscience King's College London London UK

**Keywords:** child, confirmatory factor analysis, developmental psychopathology, psychometrics, psychotic symptoms

## Abstract

**Objectives:**

The current study aimed to assess the measurement invariance of the 9‐item self‐report Psychotic‐Like Experiences Questionnaire for Children (PLEQ‐C) across various demographic (age, gender, ethnicity) and psychopathology profiles in a community sample of children.

**Methods:**

Children aged 9–11 years (*n* = 613; *M* age = 10.4 years [SD = 0.8]; 50.9% female) completed questionnaire screening at school, with primary caregivers returning questionnaires by mail from home. Configural, metric, scalar, and residual invariance of the PLEQ‐C scores were investigated across groups differentiated by age (9; 10; 11 years), gender (female; male), ethnicity (white; black; other), and by child‐reported and caregiver‐reported psychopathology (abnormal rating; not abnormal).

**Results:**

The PLEQ‐C scores demonstrated good unidimensional model fit. Full configural, metric, scalar, and residual invariance were demonstrated across gender, ethnicity, and psychopathology (both child‐ and caregiver‐reported). Across age groups, the PLEQ‐C scores showed full configural and metric invariance, but only partial scalar and residual invariance (with a single item measuring differently among 11‐year‐olds).

**Conclusions:**

In this community sample, the PLEQ‐C was robust to age, gender, ethnicity, and psychopathology profiles, providing evidence of its capacity to identify children in the general population who might benefit from further assessment to determine the clinical significance of their psychotic experiences.

## 1 INTRODUCTION

Psychotic‐like experiences (PLEs; also termed subclinical psychotic symptoms or psychotic experiences) are hallucinations and/or delusions occurring in the absence of a diagnosable psychotic disorder. These experiences are particularly common during childhood, with a meta‐analysis indicating a median prevalence rate of 17% among children aged younger than 13 years (Kelleher, Connor, et al., 2012). While they are not pathognomonic for disorder, PLEs may be accompanied by distress and/or impairment in functioning (Dominguez et al., 2011; Linscott & van Os, 2013), and are associated with concurrent internalising and externalising psychopathology (Kalman et al., 2019; Kelleher et al., 2012b, 2014; Laurens et al., 2020). PLEs are also associated with future mental disorders, including but not limited to psychotic disorders, trauma‐ and stress‐related disorders, and anxiety disorders (Dominguez et al., 2011; Fisher et al., 2013; Kelleher, Connor, et al., 2012; Werbeloff et al., 2012). Early theory posited that PLEs might represent the behavioural expression of vulnerability for psychotic illness (an ‘extended psychosis phenotype’) regardless of age, particularly when they persist over time (van Os & Linscott, 2012). More recently, distressing PLEs in middle childhood and early adolescence have been heralded as potentially informative markers of transdiagnostic mental health concerns (Karcher, 2022). Early identification of PLEs in childhood may provide opportunities for preventive intervention to avert adverse outcomes, with reliable and valid measurement tools required to support population screening for PLEs (Laurens & Cullen, 2016).

Whereas a range of questionnaire instruments have been used to assess PLEs in adolescent samples (Kelleher, 2015), the Psychotic‐Like Experiences Questionnaire for Children (PLEQ‐C; Laurens et al., 2007; Laurens et al., 2012) is one of the few screening instruments designed for use with community samples of children aged under 13 years. The PLEQ‐C measures childhood PLEs via self‐report (9 items) among children as young as 9 years of age. Although an accompanying 10‐item caregiver‐report version is available, there is a marked discordance between rates of self‐ and caregiver‐reported PLEs, with caregivers demonstrated to contribute less valid reports of PLEs on behalf of their children (cf. self‐report by the children) in general population screening (Gundersen et al., 2019; Gutteridge et al., 2020; Kelleher et al., 2011; Laurens et al., 2007). The PLEQ‐C has demonstrated good internal consistency and reliability in a community sample of 9‐ to 11‐year‐olds (Laurens et al., 2007), excellent ordinal alpha reliability (*α* = 0.90) (Laurens et al., 2017), and good criterion and construct validity (Gutteridge et al., 2020; Laurens et al., 2012). Endorsement of a psychotic‐like experience (PLE) on the questionnaire has good specificity (78.5%), sensitivity (73.3%), and positive (PPV: 72.1%) and negative predictive values (NPV: 79.5%) for the presence of any psychotic symptom assessed via clinician interview (Gutteridge et al., 2020). The nine self‐report items load on a unidimensional latent construct distinct from internalising and externalising psychopathology constructs, with factor loadings ranging from 0.46 (moderate) to 0.74 (strong) (Laurens et al., 2012). Despite these sound psychometric properties established for this instrument in community samples aged under 13 years, the measurement invariance of the PLEQ‐C is yet to be tested, meaning that previously explored mean differences between groups (e.g., according to age, gender, or ethnicity) might represent measurement error as opposed to true differences in PLE prevalence.

Measurement invariance aims to determine the psychometric equivalence of construct structure and item parameters across groups and is a prerequisite for comparison of group means. Measurement non‐invariance indicates that a construct is being interpreted differently by groups, such that group means cannot be meaningfully compared. Measurement invariance is commonly tested using Multi‐Group Confirmatory Factor Analysis in four hierarchical steps: configural, metric, scalar, and residual invariance (Byrne, 2016; Widaman & Reise, 1997). Configural invariance refers to equivalence of the general factor structure and pattern of item‐factor loadings across groups. Metric invariance assesses the equivalence of item loading weights across groups, evaluating whether each item contributes to the latent construct to a similar degree in different groups. Scalar invariance, in the case of categorical data within the PLEQ‐C, seeks to determine whether groups shift between response categories at similar levels of the underlying construct. Establishing scalar invariance permits comparison of factor score mean differences across groups, but evaluating residual invariance (also known as strict factorial invariance) tests whether the total of the variance of the item not shared with the construct (specific variance) and the error variance are similar across groups, allowing for summed score mean comparisons (Putnick & Bornstein, 2016). Achievement of invariance across all four successive levels indicates that groups interpret the measure in a conceptually similar manner and, therefore, group mean differences on the latent construct can be compared. Establishing evidence of measurement invariance is thus particularly important when groups demonstrate mean differences in the trait of interest.

Variation in PLE prevalence across demographic strata has been demonstrated in both clinical and non‐clinical samples. With regards to age, PLEs are more prevalent among younger respondents (aged 9–12 years) compared to adolescents (aged 13–18 years) and adults (Kelleher, Connor, et al., 2012; Linscott & van Os, 2013). Research is inconsistent concerning gender differences, with some studies and meta‐analyses observing higher PLE prevalence in males (Karcher et al., 2020; Laurens & Cullen, 2016; van Os et al., 2009), in females (Karcher et al., 2014; Ronald et al., 2014), or a lack of gender differences (Dhossche et al., 2002; Johns et al., 2004; Ndetei et al., 2012). Higher rates of PLEs have been found consistently in ethnic minorities living within Western societies, including among black minority groups and migrant communities (Eilbracht et al., 2015; Karcher et al., 2020; Laurens et al., 2008; Laurens et al., 2011; Linscott & van Os, 2013).

PLE prevalence may also vary in the presence of other psychopathology. Cross‐sectional (Nishida et al., 2008; Scott et al., 2009; Wigman et al., 2012) and longitudinal (Downs et al., 2012; Lancefield et al., 2016) studies of community samples from a variety of countries have demonstrated that individuals who report psychotic symptoms are more likely to experience internalising (i.e., anxiety and depression) and externalising (i.e., conduct/opposition and attention/hyperactivity) symptoms and disorders during adolescence. In a community sample of Irish adolescents aged 11–16 years, the presence of non‐psychotic psychopathology, whether internalising or externalising symptoms, increased the odds of self‐reported psychotic symptoms (Kelleher, Keeley, et al., 2012). The association of self‐reported PLEs with other (internalising and externalising) psychopathology was similarly identified using the PLEQ‐C in a large population sample (*n* = 27,808) of Australian children aged 11–12 years (Laurens et al., 2020).

Given the measurement invariance of the PLEQ‐C has not been examined previously, it remains unclear whether differences in PLE presentation between groups in the general population with various demographic or psychopathology profiles reflect actual prevalence disparities or differential measurement functioning across groups. That is, how the PLEQ‐C items are interpreted and answered may differ according to an individual's demographic characteristics (e.g., age, gender, ethnicity) and/or the presence of other psychopathology. Confirming the consistency of measurement across groups affords robust group mean comparisons and affirms the utility of this measure for community screening of PLEs during childhood. Therefore, the present study sought to evaluate the measurement invariance of the self‐report PLEQ‐C in a community sample of children across groups differentiated by age (9 vs. 10 vs. 11 years), gender (female vs. male), ethnicity (white vs. black vs. other), and child‐reported and parent‐reported psychopathology profiles (abnormal rating vs. not abnormal). Both child‐ and caregiver‐reported total psychopathology were used to capture the unique perspectives afforded by different informants (De Los Reyes et al., 2015; van der Ende et al., 2012). In the context of previous demonstrations of satisfactory psychometric properties (reliability and validity) of the PLEQ‐C in community samples, it was hypothesised that the instrument would also demonstrate configural, metric, scalar, and residual invariance across all demographic and psychopathology groups.

## 2 METHOD

### 2.1 Participants and procedure

Data for analysis were drawn from the London Child Health and Development Study, within which self‐ and caregiver‐reported questionnaires were administered to a general population sample of children aged 9–11 years who attended government and religious primary schools in Greater London, United Kingdom (Gutteridge et al., 2023; Laurens & Cullen, 2016). Ethical approval for the study was provided by the Joint South London and Maudsley and the Institute of Psychiatry National Health Service Research Ethics Committee.

Children completed questionnaires independently and anonymously in their school classroom, supervised by researchers and the classroom teacher, with questionnaire instructions and items read aloud by a researcher. Of 8431 children invited to participate in the study, self‐report questionnaires were completed in class by 7968 (94.5%; 382 caregivers and 81 children refused consent for the child's participation).^1^ At the conclusion of the classroom administration, a caregiver‐report questionnaire (matched to the child's questionnaire by code number) was issued to each participating child for completion at home by their primary caregiver. These caregiver questionnaires were returned via reply‐paid mail for 1496 children (17.7% of those with self‐reported PLEQ‐C data; total PLEQ‐C score did not differ between children for whom caregiver information was and was not available: *M* = 6.34, SD = 4.24 and *M* = 6.42, SD = 4.15, respectively; *t*(1,494) = 0.63, *p* = 0.53]). We excluded data from 89 children who were missing responses on any item relevant to the present study (a non‐significant Little's Missing Completely at Random test suggested that these responses were missing completely at random, with age the most frequently missing item [*n* = 24]), leaving 1407 children with complete data.

The demographic characteristics of interest in this study of measurement invariance (age, gender, and ethnicity) were disproportionately represented within this London school‐based sample of 1407 children. As markedly unbalanced group sizes may lead to spurious determinations of invariance (Yoon & Lai, 2018), a study subsample was derived for analysis that equivalently represented the key demographic characteristics under investigation while retaining maximum data within the least‐represented demographic strata. Accordingly, disproportionate stratified random sampling was conducted to select ∼20 children within each of 36 strata differentiated by age (6‐monthly intervals between 9.00 and 11.99 years), gender (female; male), and ethnicity (white; black [including mixed black ethnicities]; and other). This yielded a large final sample of 613 children for analysis (*M* = 10.37 years, SD = 0.76 years, 50.9% female), with some underrepresentation (<20 children) occurring in strata located at the age extremes due to screening of children during the academic year (detailed in Supplementary Table S1). The selected (*n* = 613) and unselected (*n* = 794) samples did not differ significantly according to mean total PLEQ‐C score (Supplementary Table S2).

### 2.2 Measures

#### 2.2.1 Psychotic‐like experiences

Children completed the 9‐item self‐report PLEQ‐C (Laurens et al., 2007), which measures two hallucination‐ and seven delusion‐like experiences (detailed in Table 1) on a 3‐point response scale: *not true* (scored 0), *somewhat true* (1), and *certainly true* (2). Items were summed to obtain a total PLE score (range: 0–18).

**TABLE 1:** Item descriptive statistics for the Psychotic‐Like Experiences Questionnaire for Children (PLEQ‐C).

Item (Abbreviated item name)		Descriptive statistic		Response prevalence (%)		
		*M*	SD	NT	ST	CT
(1)	Some people believe that their thoughts can be read. Have other people ever read your thoughts? (Thoughts read)	0.48	0.64	53.9	37.0	9.1
(2)	Have you ever believed that you were being sent special messages through the television? (Special messages)	0.37	0.64	72.5	17.5	10.0
(3)	Have you ever believed that you were being followed or spied upon? (Spied upon)	0.95	0.77	28.3	38.4	33.3
(4)	Have you ever heard voices that other people could not hear? (Heard voices)	0.96	0.83	34.3	30.3	35.4
(5)	Have you ever felt that you were under the control of some special power? (Controlled)	0.80	0.75	67.5	19.3	13.2
(6)	Have you ever known what another person was thinking even though that person wasn't speaking? (Read minds)	0.67	0.76	35.4	40.3	24.3
(7)	Have you ever felt as though your body had been changed in some way that you could not understand? (Body changed)	0.47	0.72	51.1	25.8	23.1
(8)	Do you have any special powers that other people don't have? (Special powers)	0.63	0.79	58.7	21.4	19.9
(9)	Have you ever seen something or someone that other people could not see? (Seen things)	0.78	0.82	45.8	24.5	29.7

Abbreviations: CT, Certainly True; NT, Not True; ST, Somewhat True.

#### 2.2.2 Total psychopathology (internalising and externalising)

Children and caregivers, respectively, independently completed the 25‐item self‐ and parent‐report versions of the Strengths and Difficulties Questionnaire (SDQ; Goodman, 1997; Goodman et al., 2003), which is a brief behavioural screening questionnaire for children aged 3–16 years. The SDQ measures prosocial behaviour and four domains of childhood psychopathology (emotional symptoms, peer relationship problems, conduct problems, and hyperactivity‐inattention). There are five items per subscale, each rated on a 3‐point response scale: *not true* (scored 0), *somewhat true* (1), and *certainly true* (2). A Total Difficulties score, which indexes overall (internalising and externalising) psychopathology, is calculated by summing items from the four psychopathology subscales (range: 0–40), with these scores evidencing good criterion validity against clinical diagnoses of childhood mental disorder (Goodman & Goodman, 2011). According to population norms specified for the SDQ (Goodman et al., 2003), the current study demarcated children scoring in the ‘Abnormal’ range of the Total Difficulties scale (comprising approximately 10% of children with the poorest functioning in population samples) from the remaining children—designated here as the ‘Not abnormal’ group—who scored in the ‘Normal’ (1^st^‐∼80^th^ centile) or ‘Borderline’ (∼81^st^–90^th^ centile) ranges. For the self‐report version in community samples, satisfactory internal and test‐retest reliability have been demonstrated from as young as 8 years of age (Goodman, 2001; Goodman et al., 2003; Mellor, 2005; Muris et al., 2004) and construct validity from 9 years (Hobbs & Laurens, 2020). Sound inter‐rater reliability, internal reliability, and test‐retest reliability for the caregiver‐report version has also been established (Goodman, 2001; Goodman et al., 2003; Mellor, 2004).

### 2.3 Statistical analysis

Descriptive statistics were calculated for the nine PLEQ‐C items using IBM SPSS (version 28; IBM Corp, 2021). Successive MG‐CFAs were then conducted in Mplus (version 8; Muthén & Muthén, 2017) to evaluate measurement invariance across groups, using the largest group (in the larger study population) as the reference group in all analyses. Groups were differentiated on the basis of: (i) age (9 vs. 10 [reference group] vs. 11 years); (ii) gender (female [reference group] vs. male); (iii) ethnicity (white [reference group] vs. black vs. other); and (iv) total psychopathology (abnormal vs. not abnormal [reference group]), separately for child‐ and for caregiver‐report.

The robust Mean‐adjusted Weighted Least Square method (WLSMV) was employed for parameter estimation due to the categorical nature of the data. Theta parameterisation was utilised to prevent the constraint of means and, thereby, to permit accurate analysis where groups may differ in the mean of a latent variable (Li, 2014; Wells, 2021). Multiple goodness‐of‐fit indices were used to determine measurement invariance, including the Comparative Fit Index (CFI), Tucker‐Lewis Index (TLI), and Root Mean Square Error of Approximation (RMSEA). Comparative Fit Index and TLI values of ≥0.95 indicated good fit, and values of ≥0.90 satisfactory fit (Brown, 2015; Hu & Bentler, 1999; Shek & Yu, 2014). Root Mean Square Error of Approximation values of ≤0.05 indicated good fit, and values ≤ 0.11 reasonable fit (Brown, 2015; Hu & Bentler, 1999; Shek & Yu, 2014). The Weighted Root Mean Square Residual (WRMR) is an experimental fit statistic where values of ≤1.0 signify acceptable fit (DiStefano et al., 2018); however, due to its sensitivity to sample size, the WRMR was considered secondary to other fit statistics when interpretating models. Internal consistency of the PLEQ‐C was indexed using McDonald's *ω*, which estimates true score variance as a function of item factor loadings, thus acknowledging heterogenous relations between items and avoiding assumptions of invariable means and variances (Crutzen & Peters, 2017; Dunn et al., 2014; Geldhof et al., 2014).

Measurement invariance was examined using a hierarchical series of models, beginning with establishing a well‐fitting multi‐group baseline model and then sequentially constraining model parameters (including factor loadings, item thresholds, and item residual variances) to be equal across groups (Byrne, 2016; Shek & Yu, 2014). With progressively imposed constraints, the analysed models were nested such that model fits could be evaluated by comparing goodness‐of‐fit indices between models, or the respective chi‐square fit statistic, referred to as the DIFFTEST procedure (Δ*χ* ^2^) for categorical data (Cheung & Rensvold, 2002; Meade et al., 2008; Wells, 2021). As Δ*χ* ^2^ is limited by sensitivity to sample size, Cheung and Rensvold (2002) recommend an alternative process of using ΔCFI (the difference between the CFIs of the more and the less restrictive models) to assess the practical equivalence (or nontriviality of the fit) of nested models. According to Cheung and Rensvold (2002), where the ΔCFI is < 0.01 or indicates an improved fit (i.e., a negative ΔCFI), then invariance is deemed trivial, regardless of a significant Δ*χ* ^2^, and analysis may proceed. In the current study with a large sample, Δ*χ* ^2^ was used as a primary indicator of model misfit, but where Δ*χ* ^2^ demonstrated a significant misfit, the ΔCFI was used to determine the practical significance of the misfit. Where ΔCFI was greater than 0.01 and the decrease in nested models thus considered nontrivial, then a partial invariance model was tested. This involved freeing individual item parameters to differ across groups in a stepwise manner, consistent with the order of the modification indices specified, until invariance was achieved. The four‐step measurement invariance process proceeded separately for each of the four comparisons of interest (age, gender, ethnicity, and total psychopathology), as follows:

1. *Configural invariance* involved specifying and testing the unidimensional model separately for each group. Excluding the referent/anchor item used to establish the scale of the latent variable (item 1 [Thoughts read]), the configural invariance model imposed no parameter restrictions. Configural invariance was demonstrated when the model fit the data sufficiently for each group, signifying consistent dimensional structure across groups.
2. *Metric invariance* was tested by imposing equality constraints on factor loadings across groups (equal to the reference group) and then comparing model fit to the baseline (configural) model in which no constraints (excepting to the referent item) were applied. If the metric invariance model fit was significantly and practically poorer relative to the baseline (configural) model, then metric invariance was not substantiated, indicating at least one factor loading differed between groups.
3. *Scalar invariance* was explored by maintaining the prior constraints on factor loadings from the metric model and additionally constraining item thresholds. If the model fit obtained with the constrained item thresholds was significantly and practically worse than that obtained for the metric model, then scalar invariance was not supported. As full scalar invariance is not essential for meaningful analysis, partial scalar invariance may be sought in instances where at least two items per construct are equivalent (Byrne et al., 1989). Partial invariance models were tested by consulting modification indices and progressively freeing relevant individual item parameters until the change from metric invariance became non‐significant or the ΔCFI trivial.
4. Lastly, *residual invariance* was tested by constraining item residuals to be equivalent (fixed to 1.0) across groups and then comparing the constrained model fit to that obtained in a baseline model with freely estimated residuals. Residual invariance was supported when the overall model fit was not significantly worse than the freed (baseline) residual model, implying equality across groups in item residuals. Notably, due to the theta parameterisation constraining residual variances to 1.0 for nested models, the residual invariance model was identical to the scalar invariance model.

## 3 RESULTS

Descriptive statistics for each PLEQ‐C item are presented in Table 1, and sample characteristics according to the various demographic and psychopathology groups are displayed in Table 2. Mean total PLEQ‐C score (out of 18) for each demographic and psychopathology group are reported in Supplementary Table S2; these ranged from a low of 5.47 for children of ‘other’ ethnicity to a high of 9.64 for children who self‐reported in the ‘abnormal’ range on total psychopathology.

**TABLE 2:** Sample characteristics according to demographic and psychopathology categories.

Demographic and psychopathology category	Sample (*n*)	Prevalence (%)
Age		
9‐year‐olds	212	34.6
10‐year‐olds	239	39.0
11‐year‐olds	162	26.4
Gender		
Female	312	50.9
Male	301	49.1
Ethnicity		
White	226	36.9
Black	209	34.1
Other	178	29.0
Total psychopathology^a^		
Child‐report		
Not abnormal	547	89.2
Abnormal	66	10.8
Caregiver‐report		
Not abnormal	559	91.2
Abnormal	54	8.8

^a^ Measured by the Total Difficulties scale of the Strengths and Difficulties Questionnaire (Goodman, 1997).

### 3.1 Unidimensional model fit

**FIGURE 1:**
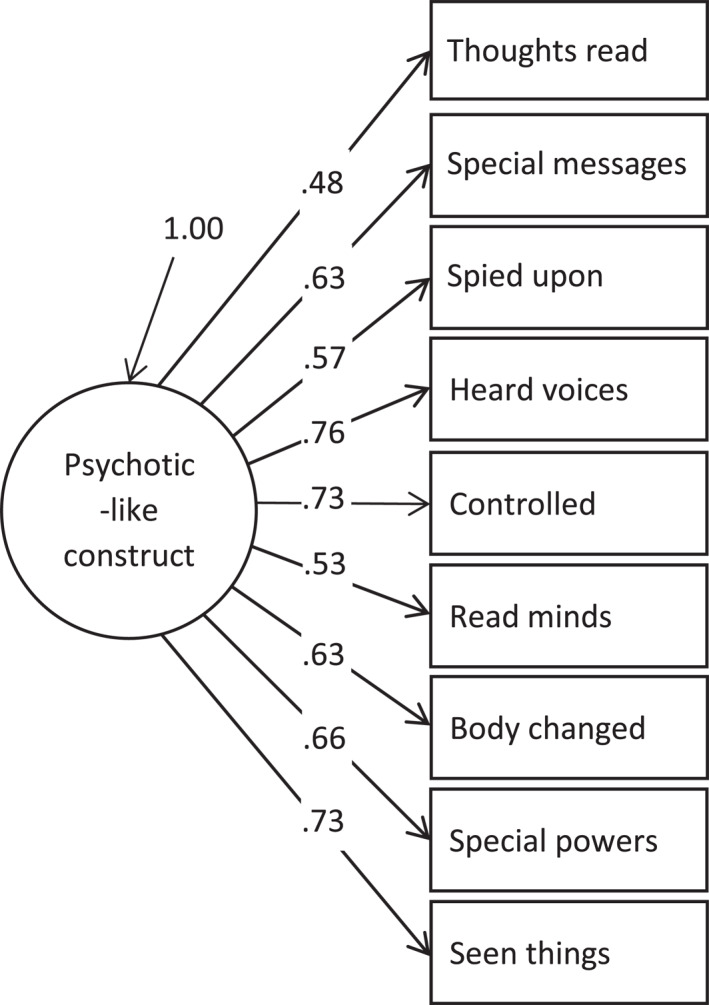
Unidimensional model derived from the confirmatory factor analysis of the Psychotic‐Like Experiences Questionnaire for Children items.

The confirmatory factor analysis of the PLEQ‐C scores explained 41.34% common variance and demonstrated good model fit for the unidimensional model (CFI = 0.987, TLI = 0.982, RMSEA = 0.041). Figure 1 illustrates the unidimensional model, with factor loadings ranging from 0.48 (moderate) to 0.76 (strong). The scale evidenced good internal consistency (McDonald's *ω* = 0.802).

### 3.2 Measurement invariance testing

The results of measurement invariance testing for the PLEQ‐C scores, examined across age, gender, ethnicity, and total psychopathology (separately by child‐ and caregiver‐report), are presented in Table 3.

**TABLE 3:** Configural, metric, scalar, and residual invariance testing of the Psychotic‐Like Experiences Questionnaire for Children (PLEQ‐C) by age, gender, ethnicity, child‐reported total (internalising and externalising) psychopathology, and caregiver‐reported total (internalising and externalising) psychopathology.

	Model Fit Information					DIFFTEST		
	RMSEA (90% CI)	CFI	∆CFI	TLI	WRMR	*χ* ^2^	df	*p*
Age								
Configural model								
9 years old	0.049 (0.010, 0.078)	0.980	‐	0.973	0.688	‐	‐	‐
10 years old^a^	0.025 (0.000, 0.059)	0.995	‐	0.993	0.619	‐	‐	‐
11 years old	0.053 (0.000, 0.087)	0.982	‐	0.976	0.678	‐	‐	‐
Configural invariance	0.041 (0.018, 0.060)	0.986	‐	0.982	1.149	‐	‐	‐
Metric invariance	0.033 (0.000, 0.052)	0.990	−0.004	0.989	1.352	16.579	16	0.413
Scalar invariance	0.041 (0.023, 0.056)	0.979	**0.011**	0.983	1.635	**58.155**	**32**	**0.003**
Partial scalar^b^	0.033 (0.005, 0.049)	0.987	0.003	0.989	1.536	37.344	30	0.167
Residual model	0.034 (0.003, 0.051)	0.988	‐	0.988	1.365	‐	‐	‐
Residual invariance^c^	0.033 (0.005, 0.049)	0.987	0.001	0.989	1.536	22.575	18	0.208
Gender								
Configural model								
Male	0.048 (0.021, 0.071)	0.983	‐	0.977	0.726	‐	‐	‐
Female^a^	0.033 (0.000, 0.059)	0.991	‐	0.988	0.657	‐	‐	‐
Configural invariance	0.040 (0.020, 0.058)	0.987	‐	0.983	0.983	‐	‐	‐
Metric invariance	0.023 (0.000, 0.043)	0.995	−0.008	0.995	1.025	2.194	8	0.975
Scalar invariance	0.022 (0.000, 0.041)	0.994	0.001	0.995	1.136	18.507	16	0.295
Residual model	0.029 (0.000, 0.047)	0.991	‐	0.991	1.087	‐	‐	‐
Residual invariance	0.022 (0.000, 0.041)	0.994	0.003	0.995	1.136	5.256	9	0.812
Ethnicity								
Configural model								
White^a^	0.045 (0.000, 0.074)	0.986	‐	0.982	0.671	‐	‐	‐
Black	0.051 (0.013, 0.080)	0.978	‐	0.970	0.713	‐	‐	‐
Other	0.049 (0.000, 0.082)	0.977	‐	0.969	0.697	‐	‐	‐
Configural invariance	0.047 (0.027, 0.065)	0.982	‐	0.976	1.205	‐	‐	‐
Metric invariance	0.039 (0.016, 0.057)	0.985	−0.003	0.984	1.415	18.143	16	0.316
Scalar invariance	0.038 (0.018, 0.053)	0.981	0.004	0.985	1.617	40.945	32	0.134
Residual model	0.036 (0.012, 0.053)	0.986	‐	0.986	1.421	‐	‐	‐
Residual invariance	0.038 (0.018, 0.053)	0.981	0.005	0.985	1.617	28.134	18	0.060
Total psychopathology (child‐report)^d^								
Configural model								
Not abnormal^a^	0.047 (0.031, 0.063)	0.981	‐	0.974	0.853	‐	‐	‐
Abnormal	0.028 (0.000, 0.102)	0.992	‐	0.989	0.593	‐	‐	‐
Configural invariance	0.041 (0.022, 0.058)	0.984	‐	0.979	1.039	‐	‐	‐
Metric invariance	0.041 (0.022, 0.057)	0.982	0.002	0.980	1.233	13.632	8	0.092
Scalar invariance	0.044 (0.029, 0.058)	0.973	0.009	0.976	1.452	**35.274**	**16**	**0.004**
Residual model	0.044 (0.028, 0.059)	0.977	‐	0.976	1.290	‐	‐	‐
Residual invariance	0.044 (0.029, 0.058)	0.973	0.004	0.976	1.452	18.710	9	0.028
Total psychopathology (caregiver‐report)^d^								
Configural model								
Not abnormal^a^	0.042 (0.025, 0.058)	0.986	‐	0.982	0.791	‐	‐	‐
Abnormal	0.056 (0.000, 0.125)	0.945	‐	0.927	0.646	‐	‐	‐
Configural invariance	0.040 (0.019, 0.057)	0.986	‐	0.982	1.022	‐	‐	‐
Metric invariance	0.028 (0.000, 0.046)	0.992	−0.006	0.991	1.082	4.790	8	0.779
Scalar invariance	0.024 (0.000, 0.042)	0.993	−0.001	0.993	1.189	15.792	16	0.468
Residual model	0.032 (0.008, 0.049)	0.988	‐	0.988	1.170	‐	‐	‐
Residual invariance	0.024 (0.000, 0.042)	0.993	−0.005	0.993	1.189	3.039	9	0.963

*Note*: Bold, underlined font demarcates the few instances in which non‐invariance was suggested via a significant DIFFTEST, thus requiring additional consultation of the ∆CFI to determine the presence of non‐invariance.

Abbreviations: CFI, Comparative Fit Index; CI, Confidence Interval; df, Degrees of freedom; RMSEA, Root Mean Square Error of Approximation; TLI, Tucker‐Lewis Index; WRMR, Weighted Root Mean Square Residual; *χ* ^2^, Chi‐square.

^a^ Reference group (the largest group in the larger study population).

^b^ Item 6, first and second thresholds freed in 11‐year‐old model.

^c^ Residual model compared to partial scalar model (Item 6, first and second thresholds freed in 11‐year‐old model).

^d^ Measured by the Total Difficulties scale of the Strengths and Difficulties Questionnaire (Goodman, 1997).

Age‐group comparisons indicated all three configural models, and the metric invariance model, achieved good fit. Metric invariance was supported by the non‐significant increase in model misfit between the configural and metric models. The scalar invariance model demonstrated a significant and nontrivial increase in model misfit, according to the DIFFTEST comparison statistic and alternative fit statistics (ΔCFI = 0.011). Guided by the largest modification indices suggested to improve model fit, the two thresholds for item 6 (Read minds) were freed in the model for 11‐year‐olds. The first threshold, indexing the level of the underlying psychotic‐like construct at which participants had a greater probability of reporting ‘somewhat true’ as opposed to ‘not true’, was constrained for the 9‐year‐old group (−0.27) at the 10‐year‐old (reference group) threshold, and freed for the 11‐year‐old group (−0.81). Likewise, the second threshold, indexing the degree of the psychotic‐like construct at which individuals moved from greater likelihood of responding ‘somewhat true’ to greater likelihood of responding ‘certainly true’, was constrained for the 9‐year‐old group (1.04) at the 10‐year‐old threshold, and freed for the 11‐year‐old group (0.66). This partial scalar invariance model returned good fit indices, with a non‐significant increase in model misfit (relative to the metric invariance model) and a ΔCFI lower than 0.01 (ΔCFI = 0.003). Full residual invariance for age could not be achieved because item parameters had been freed in the scalar model (i.e., the first and second thresholds for item 6) and these were therefore also freed within the residual models. However, the non‐significant Δ*χ* ^2^ obtained when comparing model fit between the freed baseline residual model and the residual invariance model supported partial residual invariance. (In a supplementary analysis conducted with item 6 removed, full residual invariance was achieved with the remaining 8 items; see Supplementary Table S3).

Findings for the group comparisons by gender, ethnicity, and total psychopathology (both by child‐ and by caregiver‐report) all revealed full measurement invariance. All configural models were found to have acceptable fits, indicating configural invariance, and all metric invariance models demonstrated non‐significant model misfit statistics. For gender, ethnicity, and caregiver‐reported total psychopathology, the scalar invariance and residual invariance models also demonstrated non‐significant increases in model misfit. Notably, while the child‐reported total psychopathology scalar invariance model had a significantly worse model fit (according to DIFFTEST) relative to the metric invariance model, invariance was accepted because the ∆CFI did not exceed the criterion of 0.01 (∆CFI = 0.009). Moreover, this scalar invariance model demonstrated good model fit and no modification indices were suggested. Finally, while the constrained residual invariance model for child‐reported total psychopathology demonstrated significantly worse fit than the freed baseline residual model, it nonetheless featured good fit indices and a ∆CFI less than 0.01, indicating residual invariance.

## 4 DISCUSSION

Psychotic‐Like Experiences Questionnaire for Children scores in this community sample exhibited acceptable measurement invariance for the assessment of PLEs by self‐report during middle childhood across age (9, 10, and 11 years), gender (female, male), ethnicity (white, black, other), child self‐reported psychopathology (abnormal, not abnormal), and caregiver‐reported psychopathology (abnormal, not abnormal). For the most part, results aligned with the hypotheses of full invariance in all models: full invariance was demonstrated according to gender, ethnicity, and both child‐ and caregiver‐report total psychopathology, with partial (scalar and residual) invariance demonstrated for age only. Thus, the PLEQ‐C items were similarly interpreted and responded to by children differentiated according to gender, ethnicity, and psychopathology. And, though psychometric performance was somewhat less robust across age groups than these other factors, this partial scalar and residual invariance nonetheless attests to the capacity of PLEQ‐C scores to support the population screening of children aged 9–11 years by self‐report and evaluate mean differences in PLEs across groups differentiated on demographic characteristics and psychopathology.

To our knowledge, this is the first study to examine the measurement invariance across age of a PLE questionnaire for pre‐adolescent children. Whereas configural and metric invariance were achieved across age groups, partial scalar invariance and residual invariance were achieved by allowing the first and second thresholds for item 6 (Read minds) to differ for 11‐year‐olds (with full invariance achieved when item 6 was removed). The first threshold (between responses ‘not true’ and ‘somewhat true’) and second threshold (between responses ‘somewhat true’ and ‘certainly true’) were lower in the 11‐year‐old group when allowed to vary, indicating 11‐year‐olds were more inclined to report ‘somewhat true’ and ‘certainly true’ responses at lower levels of the psychotic‐like construct than 9‐ and 10‐year‐olds. As the upper half of the 11‐year‐old group (i.e., 11.50–11.99 years) was under‐sampled, these effects might have been more prominent with greater sampling in this stratum. We speculate that the wording of item 6 (‘*Have you ever known what another person was thinking even though that person wasn't speaking?*’) could be interpreted by older children (who have greater social experience and better developed theory of mind and social competence than younger children) as reading a social cue (Goldstein & Winner, 2012). Moreover, this item, although assessing an unusual belief associated with thinking, assesses a phenomenon other than the symptoms of thought echo, insertion, withdrawal, or broadcasting (i.e., where one's own thoughts, rather than another's, are manipulated) that are typical of psychotic illness. Therefore, item 6 should be interpreted with caution or could be omitted from analyses when age is of focal interest. As Lång et al. (2020) previously discussed with regards to their demonstration of partial invariance of the 21‐item Prodromal Questionnaire‐Brief version (PQ‐B; Fonseca‐Pedrero et al., 2018) in an adult sample, the practical impacts of the differential functioning of item 6 may be diluted when using the entire PLEQ‐C.

Full residual measurement invariance was achieved across gender, replicating the trend among studies of self‐report questionnaires measuring related phenomena in older samples. Configural, metric, and scalar invariance across gender was demonstrated previously for the 21‐item PQ‐B (Fonseca‐Pedrero et al., 2018), the 92‐item Youth Psychosis at Risk Questionnaire‐Brief (Fonseca‐Pedrero et al., 2017), and the 10‐item Community Assessment of Psychic Experiences Positive Scale (CAPE‐P10; Aloba & Opakunle, 2020), in samples with mean ages of 16.13, 16.12, and 15.15 years, respectively. In one of the only other studies to analyse residual invariance, Sun et al. (2020) assessed the CAPE‐P15 (a 15‐item short‐form version of the CAPE‐P; Capra et al., 2013) and found support for the four levels of measurement invariance in their sample (*M* = 18.8 years). In a similarly aged sample to that of the present study (9–11 years; *M* = 10.0 years), the child version of the PQ‐B also demonstrated residual measurement invariance across gender (Karcher et al., 2018, 2020). Thus, it appears gender has limited impact on interpretation of PLE questionnaire items in childhood and early adolescence. This evidences a solid basis for interpreting the mean differences in PLE scores by gender that have been observed in some previous studies.

Psychotic‐Like Experiences Questionnaire for Children scores demonstrated residual invariance across ethnicity, aligning with most findings for measures of similar phenomena in other samples. Configural, metric, and *partial* scalar invariance of the PQ‐B were reported across Asian, Hispanic, white, and multiracial ethnic groups of undergraduate university students aged 17–35 years in the United States (Cicero et al., 2019). Lång et al. (2020) reported *partial* scalar invariance of the PQ‐B across ethnic minority status (white Caucasian or not) in a sample of North American undergraduate university students (*M* = 19.8 years). Among child samples, full residual invariance across African American, Hispanic, white, and other ethnicities in the United States was demonstrated for the PQ‐B child version in 9‐ to 10‐year‐olds by Karcher et al. (2018) and in a larger replication study of 9‐ to 11‐year‐olds (Karcher et al., 2020). The discrepancy between the full invariance obtained for the PQ‐B child version and the partial scalar invariance for the PQ‐B, according to ethnicity, might reflect more prominent differences between ethnicities emerging in adulthood or differences in item wording between child and adult versions. This should be considered in future research if the PLEQ‐C is adapted for use in adults.

To our knowledge, no other PLE questionnaire has been evaluated for potential interpretative differences between children with and without concurrent psychopathology symptoms or disorders. Only partial scalar invariance of the PQ‐B in a young adult undergraduate sample was previously demonstrated for depression symptoms specifically (Lång et al., 2020). Our findings indicate that children interpreted the PLEQ‐C items similarly irrespective of the presence of other internalising and externalising psychopathology. That is, neither self‐reported nor caregiver‐reported total psychopathology was associated with differential interpretation of PLEQ‐C items, with full residual invariance achieved for reports from either informant (despite cross‐informant correspondence of self‐ and parent‐reports being typically in the region of only ∼0.3 for childhood psychopathology; De Los Reyes et al., 2015; van der Ende et al., 2012). This finding of full invariance across psychopathology is important for robustly screening population samples, as the presence of PLEs comorbid with other (internalising or externalising) psychopathology may increase risk for progression to psychosis relative to PLEs alone (Binbay et al., 2011; Dominguez et al., 2011).

This study used disproportionate stratified sampling to devise an analytic sample comprising equivalent representation of participants according to demographic characteristics of interest. However, the nature of school (grade)‐based sampling (restricted to the academic year) resulted in the more limited data obtained from children aged 9.00–9.49 years and children older than 11.50 years in the sample being highly likely to be represented in the analytic sample. Despite this, no significant mean differences were found on total PLE scores between the selected and unselected samples for each demographic category. Furthermore, even after conducting disproportionate stratified sampling, the sample size was large enough to provide sufficient power to explore any potential differences between groups. Other limitations of this study include the restricted sample age range (9‐ to 11‐year‐olds only). Though the PLEQ‐C was purposively designed to elicit self‐reports from children as young as 9 years, our test of invariance across only a 3‐year age range provides limited scope to assess potential age‐related differences in the interpretation of PLEQ‐C items. As the findings suggest that a 1‐to‐2‐year age difference results in non‐invariance for one item (item 6), assessing a greater range of age groups (into adolescence) may reveal additional instances of non‐invariance and highlight how different developmental periods may impact the interpretation of PLEQ‐C items. Thirdly, as the sample was derived from London, United Kingdom, a multicultural community, results may not be generalisable to other cultural contexts. Future research should test the invariance of the PLEQ‐C across different settings. The potential effect of language background and English language proficiency on item interpretation is another path for future exploration. The grouping of ethnicities was coarse and not necessarily representative of any single (specific) ethnicity represented within these coarse groupings. The size of the sample prohibited analyses of any additional subgroups, with the ‘other’ ethnicity group being particularly heterogenous. Similarly, subthreshold psychopathology, classified as ‘borderline’ by the SDQ, was combined with the ‘normal’ category to create the ‘not abnormal’ reference group for analysis. The cut‐off scores for determining group allocation were determined from population norms (Goodman et al., 2003); however, as for all thresholds, there will likely be clinically significant psychopathology present within the borderline group. Investigating the potential differences between the interpretations of PLEQ‐C items by individuals in the normal and borderline groups would be valuable to better understand any effects of subthreshold symptom profiles. Lastly, the functional impairment or distress that may be associated with children's PLEs were also not considered and may be an avenue for future measurement invariance research exploring the comparability and cross‐symptomatic equivalence of the PLEQ‐C across these indicators of PLE impact.

In the present study, the PLEQ‐C equivalently measured PLEs across a range of demographic and psychopathology profiles in middle childhood (aged 9–11 years) in a community sample, indicating scope for robust comparison of group mean differences. Psychotic‐Like Experiences Questionnaire for Children scores exhibited configural, metric, scalar, and residual invariance for eight of nine items across age, gender, ethnicity, and both child self‐reported and caregiver‐reported total psychopathology, with only a single item demonstrating differential scalar (threshold) functioning (i.e., achieving partial invariance only) across age groups. This study endorses the use of the PLEQ‐C for screening community samples of children to identify those in the middle childhood population who report PLEs, and who might benefit from further assessment to determine the clinical significance of their psychotic experiences.

## CONFLICT OF INTEREST STATEMENT

The authors declare they have no conflicts of interest to disclose.

## Supporting information

Supporting Information S1

## Data Availability

The data that support the findings of this study are available on request from the corresponding author, subject to ethical restrictions. The data are not publicly available due to privacy or ethical restrictions.

## ENDNOTE

^1^
These data were reported previously in a structural analysis (exploratory and confirmatory factor analyses) and item‐response theory analysis of the self‐report PLEQ‐C (Laurens et al., 2012). The current study of measurement invariance of the PLEQ‐C in a community sample re‐uses data from a selected subsample (*n* = 613) of this larger sample, as described further in the text.
