# Supplementary material for: Measurement invariance across age, gender, ethnicity, and psychopathology of the Psychotic‐Like Experiences Questionnaire for Children in a community sample
Source: Int J Methods Psychiatr Res. 2023 Mar 2;32(4):e1962. doi: 10.1002/mpr.1962 (PMC10698830; doi:10.1002/mpr.1962)
Supplement: Supplementary file 1 — Supporting Information S1 [file MPR-32-e1962-s001.docx]

**Measurement invariance across age, gender, ethnicity, and psychopathology of the Psychotic-Like Experiences Questionnaire for Children in a community sample**

Lauren M. Piltz^1^, Emma J. Carpendale^1^, Kristin R. Laurens^1,2^

^1^ Queensland University of Technology (QUT), School of Psychology and Counselling, Brisbane, QLD, Australia

^2^ Department of Psychosis Studies, Institute of Psychiatry, Psychology, and Neuroscience, King’s College London, London, UK

***Supplementary Online Material***

*Supplementary Table S1:* Demographic strata containing fewer than 20 children in the final selected sample (*n*=613) 2

*Supplementary Table S2:* Comparison of the mean total psychotic-like experiences score by demographic and psychopathology comparison groups in the selected (*n*=613) and unselected (*n*=794) samples 3

*Supplementary Table S3.* Configural, metric, scalar, and residual invariance testing of eight items of the Psychotic-Like Experiences Questionnaire for Children (PLEQ-C) by age (excluding item 6) 4

Supplementary Table S1. Demographic strata containing fewer than 20 children in the final selected sample (*n*=613)

| Demographic strata by age (in years), gender, and ethnic group | Available *n* |
| --- | --- |
| 9.00-9.49, male, black | 18 |
| 9.00-9.49, male, other | 11 |
| 9.00-9.49, female, black | 19 |
| 9.00-9.49, female, other | 14 |
| 9.50-9.99, male, white | 19 |
| 9.50-9.99, male, other | 11 |
| 10.50-10.99, male, other | 19 |
| 11.00-11.49, male, white | 19 |
| 11.00-11.49, male, other | 19 |
| 11.00-11.49, female, other | 11 |
| 11.50-11.99, male, white | 12 |
| 11.50-11.99, male, black | 8 |
| 11.50-11.99, male, other | 5 |
| 11.50-11.99, female, white | 16 |
| 11.50-11.99, female, black | 4 |
| 11.50-11.99, female, other | 8 |

Supplementary Table S2. Mean total psychotic-like experiences score by demographic and psychopathology comparison groups in the total, selected (*n*=613), and unselected (*n*=794) samples

| Comparison groups | Total sample (*n*=1,407) | Selected sample (*n*=613) | Unselected sample (*n*=794) | Comparison: selected vs. unselected | |
| --- | --- | --- | --- | --- | --- |
|  | *M (SD)* | *M (SD)* | *M (SD)* | *t* | *P* |
| **Age** |  |  |  |  |  |
| 9-year-olds | 6.87 (4.34) | 6.55 (4.30) | 7.16 (4.55) | 1.45 | .149 |
| 10-year-olds | 6.27 (4.14) | 5.92 (3.95) | 6.47 (4.25) | 1.65 | .100 |
| 11-year-olds | 5.74 (4.11) | 5.85 (4.30) | 5.62 (3.91) | -0.48 | .629 |
| **Gender** |  |  |  |  |  |
| Female | 6.33 (4.25) | 6.16 (4.17) | 6.45 (4.30) | 0.92 | .360 |
| Male | 6.35 (4.25) | 6.07 (4.18) | 6.57 (4.31) | 1.54 | .125 |
| **Ethnicity** |  |  |  |  |  |
| White | 5.99 (4.19) | 5.77 (4.21) | 6.09 (4.18) | 0.96 | .338 |
| Black | 7.11 (4.40) | 7.04 (4.33) | 7.16 (4.46) | 0.31 | .760 |
| Other | 5.59 (3.76) | 5.47 (3.75) | 6.78 (3.72) | 1.41 | .160 |
| **Total psychopathology ^a^** |  |  |  |  |  |
| Child-reported |  |  |  |  |  |
| Not abnormal | 5.92 (4.05) | 5.69 (3.97) | 6.11 (4.11) | 1.79 | .074 |
| Abnormal | 9.53 (4.39) | 9.64 (4.15) | 9.46 (4.57) | -0.26 | .801 |
| Caregiver-reported |  |  |  |  |  |
| Not abnormal | 6.15 (4.22) | 5.93 (4.16) | 6.33 (4.27) | 1.66 | .097 |
| Abnormal | 7.96 (4.12) | 8.04 (3.83) | 7.91 (4.35) | -0.18 | .859 |
| Total score | 6.34 (4.28) | 6.12 (4.17) | 6.51 (4.30) | 1.70 | .089 |
| *Note.* ^a^ Measured by the Total Difficulties scale of the Strengths and Difficulties Questionnaire (Goodman, 1997). | | | | | |

Supplementary Table S3. Configural, metric, scalar, and residual invariance testing of eight items of the Psychotic-Like Experiences Questionnaire for Children (PLEQ-C) by age (excluding item 6)

|  | Model Fit Information | | | | |  | DIFFTEST | | |
| --- | --- | --- | --- | --- | --- | --- | --- | --- | --- |
|  | RMSEA (90% C.I.) | CFI | ∆CFI | TLI | WRMR |  | Χ^2^ | df | *p* |
| Configural Model |  |  |  |  |  |  |  |  |  |
| 9 years old | .047 (.0, .081) | .985 | - | .979 | .632 |  | - | - | - |
| 10 years old^a^ | .032 (.0, .068) | .993 | - | .990 | .607 |  | - | - | - |
| 11 years old | .062 (.013, .099) | .978 | - | .969 | .668 |  | - | - | - |
|  |  |  |  |  |  |  |  |  |  |
| Configural Invariance | .044 (.018, .065) | .986 | - | .981 | 1.104 |  | - | - | - |
| Metric Invariance | .035 (.0, .055) | .990 | -.004 | .989 | 1.318 |  | 14.709 | 14 | .398 |
| Scalar Invariance | .032 (.0, .051) | .988 | .002 | .990 | 1.511 |  | 31.850 | 28 | .281 |
| Residual Model | .035 (.0, .055) | .988 | - | .988 | 1.356 |  | - | - | - |
| Residual Invariance | .032 (.0, .051) | .988 | .0 | .990 | 1.511 |  | 17.882 | 16 | .331 |
| *Note.* ^a^ Reference group (the largest group in the larger study population). | | | | | | | | | |
